# Blood-Based Surveillance Biomarkers for Gastroesophageal Cancers

**DOI:** 10.3390/cancers17213552

**Published:** 2025-11-02

**Authors:** Neda Dadgar, Muhammad Anees, Christopher Sherry, Hyun Young Park, Erin E. Grayhack, Arul Goel, Alisha F. Khan, Ashten Omstead, David L. Bartlett, Patrick L. Wagner, Ali H. Zaidi

**Affiliations:** 1 Allegheny Health Network Cancer Institute, Pittsburgh, PA 15224, USA; neda.dadgar@ahn.org (N.D.); muhammad.anees@ahn.org (M.A.); christopher.sherry2@ahn.org (C.S.); hyunyoung.park@ahn.org (H.Y.P.); erin.grayhack@ahn.org (E.E.G.); alishafkhan@icloud.com (A.F.K.); ashten.omstead@ahn.org (A.O.); david.bartlett@ahn.org (D.L.B.); patrick.wagner@ahn.org (P.L.W.); 2 Department of Molecular, Cellular & Developmental Biology, University of California Santa Barbara, Santa Barbara, CA 93106, USA; agoel@ucsb.edu

**Keywords:** liquid biopsy, circulating tumor DNA, microRNA, methylation, biomarkers, surveillance

## Abstract

**Simple Summary:**

Gastroesophageal cancers are often diagnosed late and frequently recur. Current surveillance methods are invasive and sometimes inadequate. This review examines “liquid biopsies”—blood tests that detect cancer markers like circulating tumor DNA (ctDNA), methylated DNA, and other tumor-derived substances. These tests offer a minimally invasive way to monitor patients. While ctDNA and methylated DNA show particular promise for earlier recurrence detection compared to imaging, most blood-based biomarkers are still investigational. The integration of multi-analyte assays, AI, and ongoing clinical trials suggests that liquid biopsies will soon become a transformative tool for improved surveillance and personalized care in gastroesophageal cancers.

**Abstract:**

Gastroesophageal cancers including esophageal and gastric cancer remain major causes of global cancer mortality, primarily due to late diagnosis and high recurrence rates after curative treatment. Current surveillance methods, such as endoscopy and imaging, are invasive, costly, and often inadequate for detection. Blood-based biomarkers (“liquid biopsies”) offer a minimally invasive alternative capable of real-time tumor monitoring. In this review, we summarize recent advances across all major classes of blood-derived biomarkers: circulating tumor DNA (ctDNA), methylated DNA, cell-free RNAs (microRNAs, lncRNAs, circRNAs), circulating proteins, autoantibodies, circulating tumor cells, extracellular vesicles, and metabolites. Reviewing the existing literature on gastroesophageal cancers, we highlight current evidence, validation phases, performance metrics, and limitations. Special attention is given to clinical trial evidence, including ctDNA monitoring studies, that demonstrated earlier recurrence detection compared to imaging. While blood-based biomarker analysis has not yet supplanted endoscopy as standard of care in gastroesophageal cancer surveillance, the convergence of multi-analyte assays, AI, and clinical validation trials positions liquid biopsy as a transformative tool in the surveillance of gastroesophageal cancers.

## 1. Introduction

Gastric and esophageal cancers are a significant global health burden, ranking among the top five cancers for diagnosis and mortality in 2022 [1]. Esophageal cancer also poses a substantial threat, with an estimated 511,054 new cases and 445,391 deaths globally in 2022 [2]. Their lethality stems from non-specific symptoms (nausea, pain, early satiety) that delay diagnosis, often resulting in peritoneal metastasis at presentation. This leads to poor prognoses, with 5-year survival for esophageal cancer under 25% and advanced gastric cancer survival around 12 months [3,4,5]. 

Current treatments include surgery (esophagectomy, gastrectomy), chemotherapy (neoadjuvant, adjuvant, palliative, e.g., FOLFOX, cisplatin, FLOT, CAPOX), radiation, and targeted therapies (trastuzumab for HER2+, immune checkpoint inhibitors) [6,7]. However, recurrence rates are high (40–60%), indicating a critical need for more effective surveillance strategies [8] highlights the need for more effective surveillance strategies.

There is an unmet need for minimally invasive surveillance strategies that can detect cancer recurrence earlier and more readily than conventional methods. Circulating biomarkers originate through tumor apoptosis, necrosis, and active secretion of nucleic acids and vesicles into the bloodstream. These fragments reflect tumor burden, genetic heterogeneity, and host–immune interactions, providing a minimally invasive window into disease dynamics. Blood-based biomarkers measure tumor-derived or tumor-associated factors circulating in peripheral blood, offering a promising approach for such surveillance. These “liquid biopsies” offer potential advantages, including safety, repeatability, cost-effectiveness, and patient acceptability, which enable real-time tumor monitoring with higher compliance. Earlier detection of recurrence through blood-based surveillance could enable more timely intervention with salvage therapies, leading to improved patient outcomes and potentially personalized treatment approaches. In this review, we provide an overview of the major classes of blood assays and biomarkers under investigation, detail current and emerging biomarkers for each gastroesophageal (GE) cancers subtype, esophageal squamous cell carcinoma (ESCC) vs. adenocarcinoma and gastric adenocarcinoma (EAC), and discuss their mechanisms, performance metrics, regulatory status, and limitations. Diagnostic biomarkers enable early detection, prognostic biomarkers stratify outcomes or therapeutic response, and surveillance biomarkers monitor minimal residual disease (MRD) and recurrence. This review focuses on the latter, emphasizing translational and clinical readiness. Relevant publications were identified through targeted searches of PubMed, Embase, and ClinicalTrials.gov (2010–2025) using combinations of ‘gastroesophageal cancers’, ‘liquid biopsy’, ‘ctDNA’, and ‘surveillance’.

## 2. Overview of Blood-Based Biomarker Assays and Classes

Multiple classes of blood-based biomarkers are being explored for cancer detection and surveillance. Each class offers distinct biological insights and technical considerations:

After curative-intent treatment for GE cancer, in both gastric and esophageal cancers, ctDNA is utilized as a prognostic tool to monitor for MRD or potential relapse [9]. Postsurgical ctDNA positivity is a strong indicator of recurrence, and longitudinal monitoring with highly sensitive, personalized assays can enable earlier detection of relapse compared to standard imaging methods [10]. This allows for better risk stratification: patients with ctDNA-positive results may benefit from more intensive surveillance or consideration for adjuvant therapy, while a negative result could potentially lead to de-escalation of treatment and prevent unnecessary toxicity [10]. Although several ctDNA tests are commercially available and some are FDA-approved for advanced cancer monitoring, their use for surveillance in GE cancer remains in the investigational stage [9,11].

Circulating cell-free RNA (cfRNA), which includes microRNAs (miRNAs), long non-coding RNAs (lncRNAs), and circular RNAs (circRNAs), is being investigated as a biomarker for cancer surveillance [12]. These RNA molecules are released by tumor cells and are appealing for their stability in the blood [12,13]. Since cfRNA markers often reflect oncogenic or tumor-suppressive pathways, they offer promise for monitoring cancer status, including the detection of minimal residual disease or recurrence [14]. However, despite promising research and some markers entering validation phases, no cfRNA-based blood testing is yet in routine clinical use for GE malignancies. Challenges include ensuring assay specificity, and ongoing research is necessary to fully validate its use in clinical practice [12,15].

Despite their decade-long use, soluble protein tumor markers exhibit suboptimal performance in surveillance for GE cancers due to several factors. Their limited sensitivity in detecting early recurrence, coupled with high false-positive rates due to inflammatory conditions, benign diseases, and lifestyle factors, compromises their reliability. The genetic limitation affecting CA19-9 production in Lewis-negative individuals further contributes to false negatives. The variable prognostic value and lack of standardized interpretation add to the challenges. As such, relying solely on soluble protein tumor markers for effective surveillance is insufficient, prompting research into combining them with other biomarkers to improve sensitivity and specificity [16,17]. Common examples include carcinoembryonic antigen (CEA), carbohydrate antigen 19-9 (CA19-9), and carbohydrate antigen 72-4 (CA72-4), among others [18]. Immunoassays detect these markers but generally exhibit poor sensitivity for early-stage disease [16]. Their moderate specificity can be compromised by benign conditions, resulting in false positives [16]. As a result, research is now focusing on combining protein markers with other modalities to improve detection sensitivity [16,17].

The immune system’s early recognition of tumor-associated antigens (TAAs) can trigger an autoantibody response that can be measured for cancer surveillance [19]. These autoantibodies are highly specific for cancer and stable in serum, with some being detectable months or years before a clinical cancer diagnosis [19,20]. Single autoantibodies, such as those against p53 or NY-ESO-1, are often found in only a minority of patients, so panels of multiple autoantibodies are used to increase sensitivity [20,21]. This approach is challenged by patient-specific antibody repertoires and the need to differentiate between cancer-induced antibodies and those from autoimmune conditions [20]. While multiplex autoantibody tests exist for other cancers, such as the Early CDT platform for lung cancer, none are currently standard for surveillance in GE cancers [22,23]. Autoantibody panels are currently under evaluation in clinical cohorts for esophageal and gastric cancers.

Intact cancer cells that have detached from a primary or metastatic tumor and entered the bloodstream are known as circulating tumor cells (CTCs) [24]. Their presence generally correlates with advanced disease and a worse prognosis [24]. For surveillance purposes, a persistent or rising CTC count after surgery can indicate residual disease and allow for real-time genotyping of the tumor [24,25]. However, in GE cancers, CTC assays face challenges due to technical limitations and low sensitivity, particularly in early-stage or non-metastatic disease [24,26,27]. While emerging microfluidic and size-based capture methods are improving detection, CTC-based tests are not yet standard for GE cancer surveillance, though research is ongoing [25,26,27].

Extracellular vesicles (EVs), including exosomes, are actively secreted by tumor cells and contain a payload of proteins, DNA, and RNA that reflect their cell of origin [28]. The contents of these vesicles are protected from degradation, making them stable in blood and useful as biomarkers for surveillance [29]. Tumor-derived exosomes, for instance, have been shown to contain characteristic miRNAs and proteins that can serve as biomarkers in GE cancers [30]. Exosomal DNA with cancer-specific mutations and various miRNAs have been studied for their diagnostic potential, and EVs in general contribute to intercellular communication [29,30]. However, challenges persist with the specialized equipment required for isolation and characterization, as well as the lack of assay standardization [29]. While EV-based diagnostics are not yet clinically utilized for GE malignancies, research is ongoing, with the potential for these assays to one day complement other liquid biopsy methods [29]. 

Tumor-specific methylation changes in cell-free DNA (cfDNA) serve as highly promising biomarkers for cancer surveillance [31,32]. Aberrant promoter methylation can act as a specific cancer signal in the blood, reflecting early events in carcinogenesis and holding promise for detecting premalignant or early changes [32,33,34]. cfDNA methylation markers are under active investigation for GE cancers [33,35]. For gastric cancer, candidate markers include P16, RASSF1A, RPRM, and RUNX3, among others, with some studies demonstrating strong performance [33,36]. In esophageal cancer, various methylated DNA markers, including SFRP1, TAC1, and P16, have been detected in circulation [37].

Tumors cause systemic metabolic changes detectable in blood through metabolomics [38]. Studies have shown altered metabolite levels in gastric cancer that partially normalize after tumor removal, suggesting their potential for monitoring biochemical recurrence [39].

Based on the current landscape of liquid biopsy assays, ctDNA and tumor-specific methylation changes in cfDNA appear promising for surveillance in GE cancers. ctDNA is already used as a prognostic tool and can enable earlier relapse detection, while cfDNA methylation markers show promise for detecting early cancer events. However, analytes like soluble protein tumor markers, circulating tumor cells (CTCs), circulating cell-free RNA (cfRNA), autoantibodies, extracellular vesicles (EVs) and metabolomics require further refinement of detection and validation before they can be routinely used for early detection and surveillance in GE cancers.

**Figure 1:**
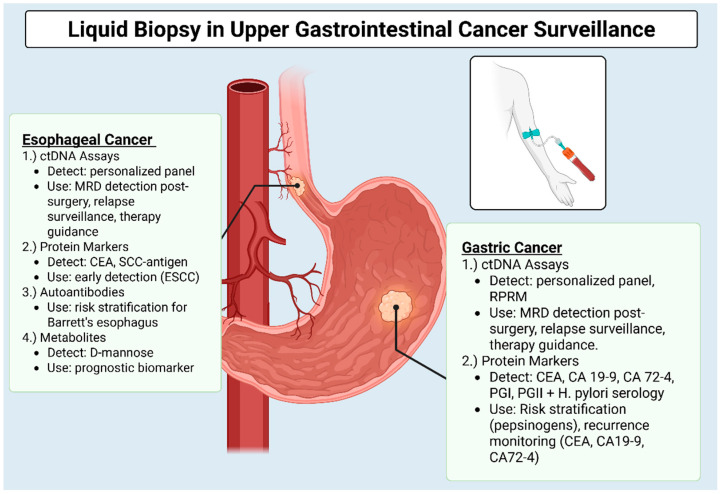
The image outlines the different types of liquid biopsy assays used for monitoring Esophageal, Gastric Cancers, along with the specific biomarkers they detect and their clinical applications (Created in BioRender. Wagner, P. (2025) https://BioRender.com/4t32mvk, accessed on 25 October 2025) Abbreviations: ctDNA: circulating tumor DNA; EAC: esophageal adenocarcinoma; MRD: minimal residual disease; miRNA: microRNA; ESCC: esophageal squamous cell carcinoma; SCC: squamous cell carcinoma; CEA: carcinoembryonic antigen; CA: carbohydrate antigen; PGI: Pepsinogen I; PGII: Pepsinogen II.

To better inform surveillance, blood-based biomarker panels are increasingly recognized as a non-invasive and comprehensive method for monitoring GE cancers. Rather than relying on a single marker, which offers limited sensitivity, combining various biomarker types such as ctDNA mutations/methylation, cancer-associated miRNAs, and protein markers allows for the capture of multiple tumor signatures. This multi-modal approach improves overall sensitivity and specificity by providing a more complete picture of tumor activity. This blood-based liquid biopsy approach offers a significant advantage for surveillance, as samples can be collected serially and less invasively than traditional biopsies, offering the potential for more frequent monitoring. In contrast, traditional surveillance methods like imaging often involve radiation exposure, logistical difficulties, and significant costs, which limit their practicality for frequent use. These limitations, combined with the inherent challenges in achieving high accuracy and precision in imaging-based tumor monitoring, can lengthen the lead time for recurrence detection. Addressing these limitations, regular, non-invasive blood-based testing enables timely and accurate monitoring of high-risk individuals and postoperative patients, offering the potential for earlier detection of recurrence or progression. Additionally, by analyzing multiple circulating components, these assays provide a dynamic assessment of a tumor’s molecular landscape over time, which could guide personalized treatment strategies and potentially improve outcomes. Comparing across biomarker modalities reveals complementary strengths. ctDNA provides high specificity for molecular relapse but limited sensitivity in early disease, whereas cfRNA and EVs capture active tumor biology. Protein and metabolite markers remain inexpensive but lack tumor specificity. Analytical limitations include cfRNA degradation, exosome isolation variability, and CHIP-related false positives. AI-assisted multi-omics pipelines integrating methylation, proteomic, and fragmentomic data represent promising directions toward clinically deployable, high-accuracy surveillance tools [40,41]. Figure 1 provides a visual overview of the different types of liquid biopsy assays used in the surveillance of GE cancers. It highlights the specific biomarkers detected by each assay type and their clinical applications in esophageal and gastric cancers. Table 1 summarizes key blood-based biomarkers evaluated across GE cancers. Among ongoing efforts, studies integrating ctDNA methylation and fragmentomics (e.g., GUIDE and GutSeer) appear particularly promising because they combine genomic and epigenetic signals with high reported sensitivity and specificity. Reported sensitivities and specificities vary widely across studies depending on stage (localized vs. metastatic), assay platform, and study design. Unless otherwise stated, metrics represent diagnostic rather than surveillance contexts. Where available, 95% CIs have been added. Together, the figure and the table provide a comprehensive overview of the current landscape of liquid biopsy biomarkers in GE cancer surveillance.

While numerous studies report promising sensitivity and specificity for blood-based biomarkers, several others have shown limited or inconsistent diagnostic utility. For instance, meta-analyses of CEA and CA19-9 in gastric cancer demonstrate substantial heterogeneity, with pooled sensitivities often below 40% due to variable assay thresholds, patient stage distribution, and ethnic differences in Lewis antigen expression. These discrepancies highlight the need for harmonized assays and prospective validation before clinical implementation [42,43]. Ethnic and environmental factors may significantly influence biomarker profiles. Variations in diet, *Helicobacter pylori* prevalence, and genetic polymorphisms (e.g., Lewis antigen phenotype affecting CA19-9) contribute to population-specific performance differences [42]. Consequently, biomarker panels validated in East Asian cohorts may not directly translate to Western populations, underscoring the need for global multicenter validation.

**Table 1:** Comparative overview of blood-based biomarker classes in gastroesophageal cancer surveillance.

Biomarker (Assay)	Cancer Type	Clinical Context	Sensitivity/Specificity	Key Analytical Limitation	Validation Phase	Source(s)
CEA (Carcinoembryonic antigen, ELISA) Class: Protein (Oncofetal antigen)	Esophageal adenocarcinoma; Gastric adenocarcinoma	Surveillance for recurrence in advanced disease (also diagnostic adjunct)	EC (detection): Se = 27.5% (18.9–35.2%) Sp = 95.4% (94.1–96.8%) Esophageal (recurrence): Se = 54.7% (40.9–67.8%) Sp = 90.0% (73.5–97.9%) GC (detection): Se = 20.1% (18.3–22.1%), Sp = 94.7% (93.6–95.7%) Gastric (recurrence): Se = 73.0% (68.8–77.2%), Sp = 59.0% (56.3–61.7%)	Low sensitivity and specificity in early-stage cancer	Esophagus: Phase 2 Gastric: Phase 3	[44,45,46,47,48,49]
CA19-9 (Carbohydrate Antigen 19-9): ELISA; Class: Protein (glycan antigen)	Gastric adenocarcinoma (subset);	Advanced disease monitoring (especially in pancreatobiliary-type or intestinal-type tumors)	GC (detection): Se = 21.4% (19.3–23.0%) Sp = 96.2% (95.2–97.1%) Gastric (Recurrence): Se = 24.1% (10.3–43.5%), Sp = 93.3% (87.3–97.1%)	Poor sensitivity, low specificity, false-negative results in some patient populations, and a lack of standardized cut-off values further hinders its reliable use	Phase 3	[48,49]
CA72-4 (Carbohydrate Antigen 72-4): ELISA; Class: Protein (glycoprotein antigen)	Gastric adenocarcinoma	Diagnostic adjunct; recurrence monitoring	Detection: Se = 58.0% (40.0–73.0%) Sp = 86.0% (80.0–90.0%) Recurrence: Se = 25.0% (0.63–80.6%) (early GC), 45.5% (30.4–61.1%) (advanced GC) Sp = 88.6% (84.6–92.0%) (early GC), 84.6% (76.9–90.4%) (advanced GC)	Low sensitivity in early stages, poor specificity and low positive predictive value	Phase 2	[50,51,52]
SCC-antigen (Squamous Cell Carcinoma Ag): ELISA; Class: Protein (squamous marker)	Esophageal squamous carcinoma	Response evaluation; recurrence surveillance in ESCC	Detection of EC: Se = 35.1% (32.0–38.3%) Sp = 95.4 (93.8–96.7%); Recurrence: Se = 26.8% (14.2–42.9%), Sp = Not reported; elevated SCC-antigen associated with poor OS	Low sensitivity and lack of diagnostic specificity due to elevation in non-malignant conditions	Phase 2	[44,53]
Pepsinogen I/II + H. pylori serology (“ABC” test) ELISA; Class: Protein enzymes (PGI, PGII) + antibody	Gastric (screening for risk)	Screening risk stratification (detects atrophic gastritis)	GC (detection): Se = 87.9% (71.8–96.6%), Sp = 50.8% (37.9–63.6%)	Low sensitivity, poor performance for early cancer, less effective for cancers of cardia and pylorus, and affected by the use of PPIs and presence of certain H pylori strains	Phase 2	[54]
Methylated Reprimo (RPRM) DNA (MSP assay) Class: ctDNA (methylated tumor DNA)	Gastric adenocarcinoma	Early detection; post-op surveillance	GC (detection): Se = 65.0% (53.5–75.3%), Sp = 75.9% (73.2–78.5%)	Heterogeneity in methylation levels across different stages of cancer, potential for false positives, and difficulty in distinguishing early-stage lesions from normal tissue	Phase 2	[55]
Circulating Tumor DNA—personalized panel (NGS) Class: ctDNA (mutations, INDELs)	Esophagus (EAC, ESCC); Gastric	MRD detection post-surgery; relapse surveillance	Esophagus/Gastric (Natera MRD detection): Se = 85.7% (69.7–95.2%) Sp = 95.5% (88.9–98.8%)	Insufficient sensitivity for very low ctDNA concentrations and high technical variability	Phase 2	[56]
Serum DSG2 (Desmoglein-2) ELISA; Class: Protein (adhesion molecule)	ESCC; EGJ adenocarcinoma	Diagnostic/prognostic marker (ESCC)	ESCC (detection): Se = 58.2% (43.2–70.8%) Sp = 84.7% (73.0–92.8%) EJA (detection): Se = 29.2% (20.6–39.5%) Sp = 90.2% (79.1–96.0%)	Low specificity, varied diagnostic accuracy based on cancer subtype and suboptimal sensitivity for early stage	Phase 1	[57]
Multi-TAA autoantibody panel Muliplex Immunoassay; Class: Autoantibodies	Esophageal adenocarcinoma (Barrett’s)	Risk stratify Barrett’s progression	Detection: Se = 53.5–64.0% Sp = 87.0–93.7%	Inter-assay variability, lack of standardization across different multiplex platforms, and the inherent variability of autoantibody responses	Phase 2	[58]
D-mannose (serum metabolite): LC-MS; Class: Metabolite (sugar)	esophageal adenocarcinoma	Prognostic biomarker (EAC)	Low levels associated with poor prognosis; insufficient literature to report performance metrics	Lack of standardized clinical assays and high pre-analytical variability	Phase 1	[59]

Abbreviations: CEA: carcinoembryonic antigen, ELISA: enzyme-linked immunosorbent assay, EC: esophageal cancer; GC: gastric cancer, CA: carbohydrate antigen, SCC: squamous cell carcinoma, ESCC: esophageal squamous cell carcinoma, PG: pepsinogen. RPRM: Reprimo, DNA: deoxyribonucleic acid, NGS: next-generation sequencing, ctDNA: circulating tumor DNA, MRD: minimal residual disease, DSG2: desmoglein-2, EGJ: esophagogastric junction, EJA: esophagogastric junction adenocarcinoma, TAA: tumor-associated antigen.

Several completed clinical trials have significantly advanced our understanding and application of blood-based biomarkers in the surveillance and management of gastric and esophageal cancers, establishing their utility across various clinical scenarios(Table 2). One notable achievement comes from the prospective multicenter NCT05431621 (China) study, which successfully developed and validated “GutSeer,” a multi-analyte ctDNA panel incorporating methylation and fragmentomics for the non-invasive detection of multiple GI cancers, including gastric and esophageal, demonstrating high diagnostic performance in an independent cohort (NCT05431621). Beyond initial detection, the predictive power of these markers for metastasis and recurrence has been rigorously investigated. For instance, NCT02159339 (Korea) identified specific DNA methylation patterns (GFRA1m and ZNF382m) as potential biomarkers for predicting gastric cancer metastasis. At the same time, NCT04830618 (Korea) highlighted the predictive value of methylation of the c-mos proto-oncogene (MOS) for metachronous recurrence following endoscopic resection of gastric neoplasms (NCT02159339, NCT04830618).

Furthermore, ctDNA has emerged as a powerful tool for detecting MRD and recurrence monitoring. However, using ctDNA for early cancer detection and surveillance faces several challenges. Cancer cells release minimal amounts of DNA into the bloodstream, making it difficult to distinguish tumor-specific mutations from the abundance of normal DNA. A negative ctDNA result does not always confirm the absence of cancer; ctDNA levels may be below the limit of detection, or the specific mutation being tested for may not be present in the patient’s tumor. Non-cancerous conditions, such as clonal hematopoiesis of indeterminate potential (CHIP) the accumulation of age-related mutations in blood cells and other inflammatory conditions can introduce mutations that mimic cancer-related mutations, leading to false-positive results [60]. Finally, ctDNA has a short half-life, and its degradation during or after sample collection can further complicate detection efforts. Studies such as NCT02887612 (China), an impactful retrospective cohort study from China, demonstrated that postoperative ctDNA positivity in resected gastric cancer patients serves as a robust predictor for recurrence and inferior survival outcomes, often providing an earlier indication of relapse than conventional imaging NCT02887612, (tumor-informed ctDNA). Similar prognostic implications of post-treatment ctDNA dynamics have been observed in esophageal cancers, including ESCC and EAC, across various international cohorts (China, ESCC ctDNA), A United Kingdom cohort study [60] without a registered NCT identifier similarly reported that postoperative ctDNA positivity in esophageal adenocarcinoma predicted relapse within 12 months, as evidenced by NCT03425058 (China), which established that dynamic changes in ctDNA and circulating tumor cells (CTCs) during neoadjuvant chemotherapy for gastric adenocarcinoma correlate well with pathological and radiological response assessments (NCT03425058). This dynamic monitoring allows for personalized therapeutic adjustments, as demonstrated by trials such as NCT02674373 (France), which show that early clearance of ctDNA during or after neoadjuvant therapy correlates with improved patient outcomes in GE adenocarcinoma. Conversely, persistent positivity indicates poorer prognoses (NCT02674373). Finally, in the context of neoadjuvant therapy for ESCC, the NCT04005170 (China) study revealed that ctDNA negativity during or post-chemoradiotherapy combined with immunotherapy is associated with higher complete clinical response rates and better survival outcomes, highlighting the potential for biomarker-guided treatment de-escalation or intensification based on response dynamics (NCT04005170). Notably, a multi-institutional phase IB trial (NCT03044613) assessing the safety and feasibility of neoadjuvant nivolumab or nivolumab-relatlimab in resectable GE cancer patients investigated the association of serial ctDNA time points with clinical outcomes [61]. This study demonstrated that negative ctDNA preoperatively, postoperatively, and after immune checkpoint inhibition induction was associated with significantly longer recurrence-free survival and overall survival [61]. Collectively, these completed trials highlight the transformative potential of blood-based biomarkers to refine surveillance strategies, personalized treatment, and ultimately enhance the clinical outcomes for patients with GE cancers.

**Table 2:** Completed Clinical Trials Evaluating Blood-Based Biomarkers in Gastroesophageal Cancers.

Trial (Study) & ID, Country	Design (Biomarkers)	Sample Size	Study Aim	Endpoints	Status/Key Findings	References
NCT05431621; China	Prospective multicenter case–control; Biomarkers: “GutSeer” (1656-locus methylation + fragmentomics ctDNA panel)	Training/validation: 1057 cancer cases vs. 1415 controls; Testing cohort: 846 patients	To develop GutSeer, a blood-based assay using DNA methylation and fragmentomics for multi-GI cancer detection (colorectal, esophagus, gastric, pancreas, and liver)	Diagnostic performance of GutSeer assay for detecting GI cancers, measured by sensitivity, specificity, and AUC in an independent validation cohort.	Completed; Overall GI cancer cohort: AUC = 92.1% Se = 81.5%, Sp = 94.4%; Gastric: Se = 90.5% Esophagus: Se = 65.2%	[62]
NCT03425058; China	Prospective single center; Biomarkers: dMMR/MSI status with dynamic evaluation of CTC and ctDNA	50	To verify the value of ctDNA and CTC as biomarkers for tumor response in the neoadjuvant chemotherapy (nCRT) treatment of locally advance gastric adenocarcinoma.	Concordance and accuracy of response evaluation results determined by ctDNA, CTCs compared with imaging and serum tumor biomarkers (CEA, CA19-9, CA72-4)	Completed; ctDNA and CTC alteration during neoadjuvant therapy is consistent with conventional histopathological grading and radiological response assessment.	[63]
NCT05227261, Vietnam	Prospective multicenter; Biomarkers: SPOT-MAS multimodal ctDNA panel (methylation, fragmentomics, copy number, end motif)	9057	To validate the clinical utility of a multimodal non-invasive ctDNA-based MCED test, SPOT-MAS	PPV, NPV, sensitivity, and specificity of the blood ctDNA test in early detection of cancers (breast, lung, gastric, liver, colorectal)	Completed; PPV = 39.5%, NPV = 99.9%, Se = 70.7%, Sp = 99.7%; performance metrics for detecting various cancer types at 12 month follow up	[13]
NCT02159339, Korea	Prospective cohort; Biomarkers: GFRA1, SRF, ZNF382 methylation alterations. P16 and E-cadherin status as well.	198	To evaluate the feasibility of predicting GC metastasis using CDH1, GFRA1, P16 and ZNF382 DNA methylation as biomarkers.	HR, PPV and NPV of recurrence/metastasis of gastric cancer based on different methylation status	Completed; GFRA1m and ZNF382m are potential biomarkers for the prediction of pN0M0 GC metastasis	[64]
NCT04830618, Korea	Prospective cohort; Biomarkers: MOS methylation	294 overall; 123 gastric cancer vs. 171 gastric dysplasia	To evaluate if MOS methylation can be used to predict metachronous recurrence after endoscopic resection of gastric neoplasms.	MOS methylation for prediction of metachronous recurrence at least 1 year after diagnosis	Completed; MOS methylation predictive (adjusted HR = 4.76) for metachronous recurrence after endoscopic resection for gastric cancer. Se = 80.0%, sp = 53.2%,	[65]
NCT02887612, China	Prospective cohort; Biomarkers: ctDNA (targeted sequencing panel of 425 cancer-related genes)	100	Predictive value of ctDNA in Early and intermediate-stage gastric cancer	Positive Predictive Value; The proportions of patients with positive serum ctDNA that have postoperative relapse	Completed; postoperative positive ctDNA, HR = 2.74 for recurrence vs. post-ACT positive ctDNA, HR = 15.0. Post ACT ctDNA, se = 77.8%, sp = 90.6% for recurrence.	[66]
NCT02674373, France	Prospective cohort; Biomarkers: ctDNA	82	ctDNA to predict response and risk stratification in gastric or GE adenocarcinoma	PFS, OS and tumor response rate	Completed; ctDNA + ve during NAT (HR = 6.2), post NAT (HR = 5.3), and after surgery (HR = 12.9) associated with worst outcomes. Early ctDNA clearance during NAT associated with better outcomes	[67]
No NCT number, China	Prospective cohort; Biomarkers: tumor-informed ctDNA (NGS)	46	To evaluate MRD detection by ctDNA and its association with clinical outcome in resected gastric cancer	DFS and OS	Completed; ctDNA + ve in post-op period associated with DFS and OS (HR = 14.78 and HR = 7.66, respectively) and preceded radiographic recurrence by a median of 6 months.	[68]
NCT04005170, China	Interventional Phase 2 open label; Biomarkers: tumor naïve ctDNA (NGS)	42	To evaluate the efficacy and safety of the combination of toripalimab (an anti-PD-1 antibody) combined with definitive CRT in locally advanced ESCC	cCR, OS, PFS, duration of response and QOL	Completed; ctDNA -ve patients had a high a cCR to those with detectable ctDNA during CRT83% vs. 39%) or post CRT (78% vs. 30%). ctDNA + ve, shorter PFS and OS.	[69]
NCT04460066, NCT05543057, China	Prospective cohort; Biomarkers: ctDNA	89	To develop a MRD profiling approach with enhanced sensitivity and specificity for detecting minimal tumor DNA from cfDNA in ESCC	pCR	Completed; MRD -ve associated with pCR in neoadjuvant, surgical, and adjuvant therapy cohort whereas MRD + ve was associated with non-pCR. All MRD -ve patients stayed progression free while 23/26 MRD + ve developed progression. Similar MRD results for radiotherapy cohort	[70]
No NCT number, China	Retrospective cohort; Biomarkers: ctDNA (NGS of 77 genes)	147	Clinical utility of longitudinal ctDNA as a prognostic biomarker in ESCC	OS and PFS	Completed; For curative surgical resection, high ctDNA (HR = 7.84) and (HR = 5.71) ctDNA alterations associated with poor OS. NAT group, post NAT ctDNA (HR = 3.16) alterations associated with poor PFS.	[71]
No NCT number, United States	Retrospective cohort; Biomarkers: ctDNA (CAPP-Seq)	45	To evaluate whether ctDNA analysis can predict recurrence in patients with localized ESCA earlier than standard-of-care imaging	Distant metastases, OS and progression	Completed; Detection of ctDNA was associated with tumor progression, metastasis, and disease-specific survival.	[72]
No NCT number, United Kingdom	Prospective cohort; Biomarkers: ctDNA (77 gene panel)	97	Prognostic potential of ctDNA dynamics in EAC, taking into account CHIP	Recurrence	Completed; ctDNA in plasma following surgery for EAC is prognostic for relapse	[60]
No NCT number, Netherlands, Sweden, and Denmark	Retrospective cohort; Biomarkers: ctDNA	42	To detect ctDNA alterations after preoperative chemotherapy and after surgery in patients with resectable gastric cancer	Recurrence	Completed; ctDNA is a predictive biomarker of patient outcome to perioperative cancer therapy and surgical resection in patients with gastric cancer.	[73]
NCT03044613, United States	Phase IB, open-label, multicenter trial; Biomarkers: ctDNA	32	To assess the safety and feasibility of nivolumab +/− relatlimab prior to chemoradiation with II/III gastro/esophageal Cancer	Safety, feasibility, OS, RFS, MPR and pCR. ctDNA association with RFS and OS.	Completed; undetectable ctDNA post-ICI induction, preoperatively and postoperatively had a significantly longer RFS and OS	[61]

Abbreviation: NCT: National Clinical Trial identifier, ctDNA: circulating tumor DNA, GI: gastrointestinal, AUC: area under the curve, dMMR/MSI: deficient mismatch repair/microsatellite instability, CTC: circulating tumor cell, nCRT: neoadjuvant chemoradiotherapy, CEA: carcinoembryonic antigen, CA: carbohydrate antigen, SPOT-MAS: Screening for the Presence of Tumor by Methylation and Size, MCED: multi-cancer early detection, PPV: positive predictive value, NPV: negative predictive value, GFRA1: GDNF family receptor alpha-1, SRF: serum response factor, ZNF382: zinc finger protein 382, GC: gastric cancer, CDH1: cadherin-1, MOS: median overall survival, HR: hazard ratio, ACT: adjuvant chemotherapy, PFS: progression-free survival, OS: overall survival, NAT: neoadjuvant therapy, NGS: next-generation sequencing, PD-1: programmed cell death protein 1, DFS: disease-free survival, CRT: chemoradiotherapy, ESCC: esophageal squamous cell carcinoma, cCR: clinical complete response, QOL: quality of life, MRD: minimal residual disease, CAPP: Cancer Personalized Profiling, ESCA: esophageal carcinoma, CHIP: clonal hematopoiesis of indeterminate potential, EAC: esophageal adenocarcinoma, RFS: recurrence-free survival, MPR: major pathological response, pCR: pathological complete response, ICI: immune checkpoint inhibitor.

## 3. Ongoing Trials

Across ongoing trials, ctDNA remains the dominant analyte evaluated for minimal residual disease detection and treatment-response monitoring. Key endpoints include ctDNA-defined recurrence-free survival and correlation with imaging. Several trials explore methylation-based and multi-analyte panels integrating cfDNA, cfRNA, and extracellular-vesicle markers. Collectively, these studies aim to establish actionable thresholds for recurrence risk and assess whether serial liquid-biopsy monitoring can complement or reduce imaging-based surveillance. Many of these studies focus on ctDNA as a tool for detecting minimal residual disease (MRD), predicting recurrence, and monitoring treatment response. Other trials are exploring the potential of ctDNA in specific clinical scenarios, such as NCT06498752 (China) (Table 3), which is evaluating whether ctDNA MRD status after radical radiotherapy can guide consolidation therapy with PD-1 inhibitors in patients with esophageal squamous cell carcinoma (ESCC). These ongoing trials are expected to provide valuable insights into the role of blood-based biomarkers in improving the management of GE cancers. The ongoing trials highlight several potential applications of blood-based biomarkers in the surveillance of GE cancers. ctDNA is being extensively investigated for MRD detection post-treatment in trials such as NCT05029869 and NCT06893133, to identify high-risk patients for adjuvant therapy or closer monitoring, and for early treatment efficacy indication through ctDNA clearance. Biomarkers, including ctDNA, are also being explored to predict treatment response in trials such as NCT04053725 and NCT06662110, with the potential to inform personalized treatment decisions. Longitudinal monitoring of biomarkers such as ctDNA and CTCs, as seen in NCT02610218, could provide real-time insights into disease progression and treatment response, allowing for earlier recurrence detection than traditional imaging. Furthermore, trials such as NCT05366881 and NCT07035587 are developing blood-based tests for early cancer detection, potentially improving outcomes through earlier intervention.

## 4. Translational Significance and Future Directions

**Figure 2:**
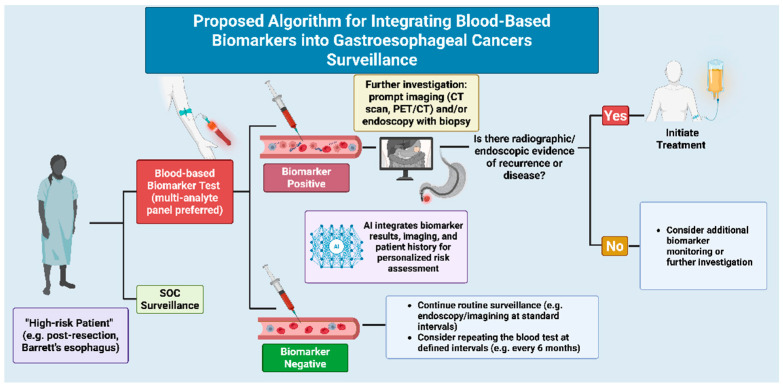
Proposed Algorithm for Integrating Blood-Based Biomarkers into GE Cancer Surveillance. This flowchart outlines a strategy for managing high-risk GE cancer patients, such as that post-resection or with Barrett’s esophagus. It integrates blood-based biomarker testing (preferably multi-analyte panels) with AI-powered personalized risk assessment, prompting further investigation (imaging and/or endoscopy with biopsy) for biomarker-positive cases to detect recurrence or disease. Negative biomarker results guide continued routine surveillance, potentially with repeat blood tests at defined intervals (Created in BioRender. Wagner, P. (2025) https://BioRender.com/gzun2g6, accessed on 25 October 2025). Abbreviations: AI: artificial intelligence, CT: computed tomography, PET/CT: positron emission tomography/computed tomography, SOC: standard of care, GE: gastroesophageal, MRD: minimal residual disease, cfDNA: cell-free DNA, ctDNA: circulating tumor DNA, EV: extracellular vesicle, miRNA: microRNA, NGS: next-generation sequencing, CA: carbohydrate antigen, CEA: carcinoembryonic antigen, ESCC: esophageal squamous cell carcinoma, EAC: esophageal adenocarcinoma.

The integration of blood-based biomarkers promises to transform GE surveillance, enabling earlier recurrence detection and less invasive monitoring (Figure 2). This strategy utilizes multi-analyte biomarker panels in high-risk patients or with Barrett’s esophagus. A positive biomarker result triggers imaging and/or endoscopy, while AI integrates biomarker data with imaging and patient history for personalized risk assessment. Confirmed recurrence prompts treatment, whereas negative results guide continued routine surveillance, potentially with serial blood tests. This approach, by reducing unnecessary invasive procedures, aims to personalize follow-up and significantly improve patient outcomes. Recent advances integrate genomics, epigenomics, proteomics, and metabolomics into multi-omics liquid biopsy assays. Multi-cancer early detection (MCED) tests such as *GutSeer* and *SPOT-MAS* exemplify this approach, detecting several GI cancers in a single assay. Despite encouraging accuracy, real-world adoption will depend on cost-effectiveness, laboratory standardization, and reimbursement frameworks. Modeling studies suggest that cost per recurrence detected must fall below imaging-based surveillance to achieve widespread implementation. Future directions include seamless integration with AI-enhanced imaging, MRD-guided therapy escalation/de-escalation, risk-stratified screening, and composite biomarker panels. Despite challenges in standardization and sensitivity, blood-based surveillance offers a more convenient and compliant approach, heralding a future where a simple blood draw guides timely curative action.

## 5. Conclusions

Liquid biopsy shows strong potential for clinical integration, but its widespread adoption will depend on large, prospective validation and demonstration of cost-effectiveness in real-world settings. Blood-based surveillance biomarkers have the potential to catch recurrences earlier (when they are still curable), reduce unnecessary invasive procedures, and personalize follow-up strategies. Combined with powerful imaging modalities and AI analytics, they promise a future where no patient with GE cancers falls through the cracks of surveillance. Artificial intelligence-driven classifiers increasingly integrate cfDNA fragmentomics, methylation, and proteomic signals using large public datasets (TCGA, ICGC) and proprietary multi-cancer training cohorts. However, validation across independent, prospectively collected cohorts remains limited, and regulatory frameworks for AI-based clinical decision tools are still evolving. Achieving this vision will require ongoing interdisciplinary collaboration and validation in clinical trials, but the foundation has been laid by the advances summarized in this review. The fight against GE cancers is poised to leverage these minimally invasive tools to improve patient outcomes and quality of life, fulfilling the urgent need that clinicians and patients alike have long recognized.

## Figures and Tables

**Table 3:** Liquid Biopsy-Based Clinical Trials for Surveillance in Gastroesophageal Cancers.

Trial (Study) & ID, Country	Design (Biomarkers)	Sample Size	Study Aim	Endpoints	Status/Key Findings
NCT05029869; Vietnam	Prospective observational; Biomarkers: ctDNA	100	Use of NGS to detect ctDNA in gastric cancer after surgery	Sensitivity/specificity of MRD detection using ctDNA	Ongoing (Active, not recruiting)
NCT06232395, China	Prospective multicenter cohort: Biomarkers: ctDNA	1197	To develop and validate a new multi-target marker early detection and postoperative monitoring of gastric cancer	Performance of the multi-target panel in diagnosis, detecting postoperative recurrence and metastasis	Ongoing, recruiting
NCT04993378, China	Prospective cohort: Biomarkers: extracellular vesicle (EV) protein signature “EV-score” derived from four EV proteins	40	Validate whether the EV-score can predict and monitor immunotherapy outcomes in advanced gastric cancer, both at baseline and during treatment	Performance of EV-score at baseline and longitudinally for prediction and monitoring of immunotherapeutic response (accuracy, sensitivity, specificity)	Unknown
NCT04053725, China	Prospective cohort; Biomarkers: ctDNA	200	Clinical utility of ctDNA in predicting the efficacy of immunotherapy for advanced gastric cancer.	The proportions of patients with positive serum ctDNA that have postoperative recurrence	Unknown
NCT06662110, China	Prospective cohort; Biomarkers: PSRscore calculated based on baseline serum immune proteomics	206	To validate the predictive value of systemic immune markers in predicting neoadjuvant treatment responses in advanced gastric cancer.	Sensitivity and specificity of PSRscore in predicting tumor regression/objective response/PFS/OS after NAT	Ongoing, recruiting
NCT03957564, China	Interventional phase II open label single group trial; Biomarkers: CTC, ctDNA and cfDNA	40	Clinical value of dynamic changes in CTC, ctDNA and cfDNA in NAT chemotherapy or and operation of resectable or locally advanced gastric or GEJ cancer	Comparison of biomarker dynamics with CT/RECIST responses and prognosis (e.g., recurrence, survival)	Unknown
NCT06893133; China	Prospective observational multicenter; Biomarkers: personalized ctDNA-MRD	110	Correlation between ctDNA-MRD status and tumor recurrence and metastasis in gastric cancer patients who have received neoadjuvant therapy followed by curative resection	Sensitivity, Specificity, and Positive predictive value of ctDNA-MRD in predicting postoperative recurrence	Ongoing (Active, not recruiting)
NCT01715233, United States	Phase 2, single arm treatment trial; Biomarkers: CHFR methylation	27	To estimate and compare the response rates in metastatic GE patients treated with mDCF based on methylation status of CHFR.	objective response rate (PR/SD/PD) stratified by CHFR methylation	Completed; results not reported
NCT06979895, China	Prospective cohort; Biomarkers: ctDNA multigene methylation panel	150	Correlate methylation dynamics with treatment response for gastric cancer	Association between changes in methylation and objective response	Ongoing; not yet recruiting
NCT06335576, China	Prospective single center cohort; Biomarkers: serum proteomics panel	89	Establish circulating proteomic subtypes of gastric cancer and explore their clinical applicability	Identification and reproducibility of serum-based proteomic subtypes (e.g., classification accuracy, subtype detection)	Ongoing; not yet recruiting
NCT02610218, China	Prospective cohort; Biomarkers: ctDNA (HER2 amplification/mutations) + CTCs	124	Evaluate whether changes in cfDNA levels and CTC counts correspond with therapeutic response to HER2-targeted therapy in metastatic HER2-positive gastric cancer	Concordance of ctDNA/CTCs with radiologic treatment response, emergence of HER2 resistance, and detection of progression	Unknown
NCT04511559, China	Prospective cohort; Biomarkers: ctDNA methylation	540	To describe ctDNA methylation profile in gastric cancer and demonstrate correlation between ctDNA methylation status and diagnosis and prognosis	Analysis of ctDNA methylation status and its correlation to early diagnosis and prognostic evaluation of gastric cancer	Unknown
NCT05513144, China	Prospective cohort; Biomarkers: ctDNA	30	To evaluate the use of next generation sequencing (NGS) to detect circulating tumor DNA in advanced HER2 negative gastric cancer patients	Prognostic molecular markers; The sensitivity and specificity of ctDNA detection	Unknown
NCT05208372, China	Prospective case–control; Biomarkers:	200	Value of CTCs and ctDNA in the diagnosis of metastasis in ascites/peritoneal flushing fluid and blood	Quantity of CTCs; Expression of ctDNA	Unknown
NCT04576858, Denmark	Prospective cohort; Biomarkers: ctDNA	1950	To evaluate the treatment effect as well as predictive and prognostic factors with special emphasis on the clinical utility of ctDNA in plasma in patients with GE cancer	Time to recurrence	Unknown
NCT05348161, China	Interventional non-randomized parallel: Biomarkers: HER2/PD-L1-positive CTCs; ctDNA genomic events	100	To evaluate how HER2-targeted therapy and immunotherapy affect molecular profiles in HER2-positive gastric cancer patients via multi-omics liquid biopsy markers	Proportions of HER2- and PD-L1-positive CTCs Incidence rates of various ctDNA genomic alterations (e.g., copy number changes, insertions/deletions)	Unknown
NCT07076979, China	Prospective case–control; Biomarkers: Metabolic markers	250	To develop and validate metabolic biomarkers for early diagnosis, prognosis, and prediction of recurrence and metastasis in gastric cancer	OS, DFS, HR, PPV and NPV	Ongoing, recruiting
NCT04000425, China	Prospective cohort; Biomarkers: AVENIO ctDNA surveillance kit	55	Evaluate ctDNA as an indicator of MRD and as a marker of adjuvant chemotherapy response after radical gastrectomy.	Disease recurrence risk; DFS; ctDNA changing to adjuvant chemotherapy response; Time of first negative ctDNA detection from positive ctDNA detection	Unknown
NCT05366881, United States	Prospective multicenter case–control; Biomarkers: Genome-wide cfDNA methylome enrichment	7000	Train and validate methylation-based ctDNA test for early cancer detection and MRD (including esophagus and gastric)	Sensitivity & specificity of assay vs. controls	Ongoing, recruiting
NCT07035587, Korea	Prospective-retrospective cohort; Biomarkers: Serial cfDNA (ctDNA), RNA, protein profiles	1200	Early diagnosis & post-treatment MRD monitoring for multiple cancers (including esophagus and gastric)	Sensitivity & specificity for cancer detection; VAF correlation with recurrence	Ongoing, recruiting
NCT05059444, United States, Germany, France, Italy and Spain	Prospective multicenter cohort; Biomarkers: Guardant reveal assay using methylated ctDNA	2020	Using a novel ctDNA approach to detect recurrence in early-stage solid tumors (including esophagus and gastric cancer)	Distant recurrence free interval. Lead time, sensitivity, and specificity and of ctDNA in detecting recurrence	Ongoing, recruiting
NCT06227728, Vietnam	Prospective multicenter cohort; Biomarkers: ctDNA using targeted sequencing and multiplex PCR approaches.	50	Assess if changes in ctDNA can predict early response to ICIs in patients with advanced-stage cancer (including gastric cancer)	Association between ctDNA dynamics and clinical response; comparison with RECIST; prognostic value of ctDNA clearance and with PFS/OS	Ongoing, recruiting
NCT04168931, Brazil	Interventional Phase II open label; Biomarkers: HER-2 positive CTCs	85	To investigate whether HER2-expressing CTCs may be suitable for prediction of response in patients with relapsed or metastatic gastric cancer who are histologically HER2-negative and treated with trastuzumab combination chemotherapy	Radiological response rate, frequency of HER 2 expression among CTCs of patients with recurrence or metastasis with negative expression in tumor tissue	Terminated, recruitment failure
NCT03023436, China	Interventional Phase III open label trial; Biomarkers: ctDNA and CTCs	220	To assess ctDNA and CTC alterations as potential biomarkers for debulking surgery combined with HIPEC and systemic chemotherapy in patients with gastric cancer and peritoneal dissemination (as a secondary outcome measure)	Median survival time, OS, PFS, morbidity and mortality, QOL, CTCs changes, ctDNA changes, and molecular biomarker (including 14 genes) alteration	Unknown status
NCT04510285, United States	Interventional Phase II open label; Biomarkers: ctDNA	48	To evaluate differences in 6-month ctDNA clearance rate in HER2+ esophagogastric cancer with persistent ctDNA following curative surgery when treated with “second adjuvant” trastuzumab with or without pembrolizumab	Rate of ctDNA clearance at 6 months	Terminated, recruitment failure
NCT04665687, Korea	Prospective cohort; Biomarkers: ctDNA	1730	To differentiate early gastric cancer and precancerous adenoma and predict recurrence by finding biomarkers through molecular profiling	Biomarker-based differentiation between adenoma and early GC; prognostic biomarkers for recurrence	Unknown status
NCT05594381, China	Interventional Phase II open label; Biomarkers: ctDNA	90	To dynamically detect gene mutations, protein expressions and tumor images in G/GEJ tumor tissues and blood samples before, under and after PD-1 monoclonal antibody (sintilimab) combined with SOX neoadjuvant therapy by using ctDNA targeted sequencing combined with multi-omics technology	pCR, ORR, DCR, MPR, TRG, R0 resection rate, OS, tumor downstaging, DFS, treatment-emergent adverse events and 30-day postoperative mortality	Not yet recruiting
NCT04929015, United States	Interventional open label; Biomarkers: tumor-informed personalized ctDNA assay (Signatera)	30	Utility of ctDNA as a sensitive biomarker in patients with Peritoneal Carcinomatosis treated with chemotherapy, CRS and/or HIPEC	Clearance rate of ctDNA with cytoreductive surgery (CRS), comparing with clinical staging of CRS and activity of chemotherapy in this disease	Ongoing, recruiting
NCT05482516, United States	Interventional Phase III open label; Biomarkers: tumor-informed personalized ctDNA-MRD assay (Signatera)	20	Guide atezolizumab and bevacizumab therapy by MRD status in GI cancer	Rate of Signatera ctDNA positivity, rate of enrollment, rate of ctDNA complete response, rate if ctDNA partial disease and rate of ctDNA progressive disease	Ongoing, recruiting
NCT05661110, China	Prospective cohort; Biomarkers: AmoyDx^®^ Master Panel (559 genes for DNA mutation and 1813 genes for RNA expression)	46	To analyze the correlation between genomic alterations, gene expression characteristics and the efficacy of HIPEC combined with PD1/PDL1 inhibitor conversion therapy in patients with peritoneal metastasis of gastric cancer	Relationship between the status, numerical changes in ctDNA during HIPEC combined with PD1/PDL1 inhibitor conversion therapy and postoperative R0 resection rate. Correlation between genomic changes of ctDNA and ORR, OS, RFA and event-free survival	Not yet recruiting
NCT04943406, Italy	Prospective cohort; Biomarkers: ctDNA	150	Prognostic role of ctDNA in patients with locally advanced gastric cancer	Impact of ctDNA (in peritoneal lavage and peripheral blood) positivity on OS and DFS	Ongoing, recruiting
NCT06253650, Italy	Interventional phase II open label; Biomarkers: ctDNA	46	To investigate the activity, efficacy and safety of trastuzumab-deruxtecan (T-DXd) plus capecitabine/5-fluorouracil as a postoperative treatment in localized/locally advanced gastric or GE junction cancer (GC/GEJC)/esophageal adenocarcinoma patients with HER2 overexpression/amplification and positive postoperative ctDNA after preoperative 5-fluorouracil plus leucovorin, oxaliplatin, and docetaxel (FLOT) regimen followed by radical surgery	ctDNA clearance, DFS, OS, metastases-free survival, and QOL	Ongoing, recruiting
NCT05494060, China	Interventional Phase II open label; Biomarkers: ctDNA	80	To assess safety and anti-tumor activity Penpulimab in combination with Anlotinib and standard chemotherapy as adjuvant treatment for ctDNA-positive G/GEJ cancer	DFS at different time points, OS and toxicity	Ongoing, recruiting
NCT05965479, United Kingdom	Interventional Phase II open label; Biomarkers: ctDNA (Signatera assay)	25	To assess the efficacy of trastuzumab deruxtecan in reducing micrometastatic disease burden in HER2 positive GEA patients who are ctDNA positive after chemotherapy and surgery	ctDNA clearance, DFS, OS, and QOL	Ongoing, recruiting
NCT05067842, United States	Prospective case–control; Biomarkers: ctDNA (Signatera assay)	30	To determine the feasibility of assessing tumor response utilizing ctDNA in patients of locally advanced esophageal and GE junction (LA-EA/GEJ) cancer	Tumor response measured by ctDNA, R0 surgical resection, OS and RFS	Withdrawn
NCT06498752, China	Interventional Phase II open label; Biomarkers: ctDNA	102	To validate whether ctDNA MRD status after radical radiotherapy can stratify prognosis and guide consolidation therapy with PD-1 inhibitors in patients with ESCC	Median PFS, OS, cancer-specific survival, toxicity, swallowing function, dynamic ctDNA changes and correlation with recurrence	Ongoing, recruiting
NCT05759325, China	Prospective cohort; Biomarkers: ctDNA MRD	100	To observe and evaluate the correlation between ctDNA-MRD and the therapeutic effect and prognosis of stage II-IVA operable ESCC	PFS rate of ESCC patients with different MRD status during perioperative period	Not yet recruiting
NCT06103890, China	Prospective cohort; Biomarkers: ctDNA	100	To explore the clinical value of MRD as a biomarker for assessing treatment efficacy, predicting recurrence risk, and evaluating prognosis in ESCC	pCR, R0 resection rate, ctDNA clearance, MPR, RFS, and OS	Ongoing, recruiting
NCT05426850, China	Prospective cohort; Biomarkers: ctDNA	100	To analyze the relationship between the dynamic changes in ctDNA and tumor relapse of ESCC treated by concurrent chemoradiotherapy	Changes in ctDNA status and recurrence, OS, and RFS.	Unknown status

Abbreviation: NCT: 2. Human Epidermal growth factor Receptor 2, HIPEC: hyperthermic intraperitoneal chemotherapy, PD1: programmed cell death protein 1, PDL1: programmed death-ligand 1, ORR: objective response rate, DCR: disease control rate, MPR: major pathological response, TRG: tumor regression grade, DFS: disease-free survival, QOL: quality of life, G/GEJ: gastric/gastroesophageal junction, ICI: immune checkpoint inhibitor, ESCC: esophageal squamous cell carcinoma, VAF: variant allele frequency, CRS: cytoreductive surgery.
